# Novel polymorphism in *FADS1* gene and fish consumption on risk of oral cancer: A case-control study in southeast China

**DOI:** 10.18632/oncotarget.15069

**Published:** 2017-02-03

**Authors:** Fa Chen, Tao Lin, Lingjun Yan, Fengqiong Liu, Jiangfeng Huang, Fangping Liu, Junfeng Wu, Yu Qiu, Lisong Lin, Lin Cai, Baochang He

**Affiliations:** ^1^ Department of Epidemiology and Health Statistics, School of Public Health, Fujian Medical University, Fujian, China; ^2^ Department of Oral and Maxillofacial Surgery, The First Affiliated Hospital of Fujian Medical University, Fujian, China

**Keywords:** oral cancer, fatty acid desaturase 1, fish consumption, polymorphism, case-control study

## Abstract

The aim of this study was to investigate the independent and combined effects of fatty acid desaturase 1 (*FADS1*) gene polymorphism and fish consumption on oral cancer. A hospital-based case-control study was performed including 305 oral cancer patients and 579 cancer-free controls. The genotypes were determined by TaqMan genotyping assay. Non-conditional logistic regression model was used to assess the effects of *FADS1* rs174549 polymorphism and fish intake. Subjects carrying A allele of rs174549 significantly reduced the risk of oral cancer (AA VS GG, OR: 0.65, 95% CI: 0.42-0.99; AA VS AG+GG, OR: 0.67, 95% CI: 0.46-0.98). Moreover, the statistically significant reverse associations were especially evident in men, smokers, alcohol drinkers and those age ≤ 60 years. Additionally, fish intake ≥7 times/week showed a 73% reduction in risk for oral cancer compared to those who ate fish less than 2 times/week (OR: 0.27, 95% CI: 0.18-0.42). Furthermore, a significant gene-diet multiplicative interaction was observed between *FADS1* rs174549 polymorphism and fish intake for oral cancer (*P*=0.028). This preliminary study suggests that *FADS1* rs174549 polymorphism and fish consumption may be protective factors for oral cancer, with a gene-diet multiplicative interaction. Functional studies with larger samples are required to confirm our findings.

## INTRODUCTION

Oral cancer is the tenth most prevalent cancer accounting for almost 300,000 new cases annually worldwide, with two thirds occurring in developing countries [1, 2]. Although tobacco smoking and alcohol drinking are major risk factors for oral cancer, there are still 15-20% non-smokers and non-drinkers developing this disease [3], indicating that genetic factors, alone or interaction with environmental factors may also be important in the development of this cancer [4, 5].

Many epidemiologic studies demonstrated that some single nucleotide polymorphisms (SNPs) were correlated with oral cancer susceptibility [6, 7]. Recently, a genome-wide association study (GWAS) identified a new susceptibility loci at fatty acid desaturase 1 (*FADS1*) gene (rs174549) associated with laryngeal squamous cell carcinoma (LSCC) in Chinese population [8]. *FADS1* encodes delta-5 desaturases and is involved in the metabolism of polyunsaturated fatty acids (PUFAs). Several SNPs at the *FADS1* gene influence the concentration of long-chain PUFAs in plasma [9]. Previous experimental studies found that PUFAs and its metabolites could inhibit tumor proliferation and invasion in head and neck cancer cell [10, 11]. However, so far, there is limited epidemiologic research on the role of *FADS1* gene polymorphism in oral cancer risk.

Fish, the major dietary sources of long chain PUFAs, is a favorite food for residents of Fujian (a province located on the southeast coast of China). The protective effect of fish intake was showed in several cancers (such as esophagus, gastric, liver, etc.)[12–14]. Edefonti et al.[15] found unsaturated fats dietary pattern could reduce the risk of oral and pharyngeal cancer. However, to date, relatively few studies have reported the fish intake related to oral cancer. Moreover, it is still unclear whether *FADS1* gene polymorphism and its interaction with fish intake could contribute to the prevention of oral cancer risk.

Therefore, the present case-control study was to assess the independent and combined effects of the new susceptibility loci (rs174549) and fish consumption on oral cancer in southeast China.

## RESULTS

The distribution of all subjects on demographic variables and potential confounding factors are described in Table 1. There were no significant differences between cases and controls in demographic characteristics (except that greater proportions of cases were lower educated and rural settings). As expected, smoking, drinking, vegetables and fruits intake were associated with oral cancer risk (*P*<0.05). The main histology types were squamous cell carcinoma (85.43%) and adenocarcinoma (8.94 %).

**Table 1:** Distribution of selected characteristics among case and control subjects

Variables	Case (%) n = 302	Control (%) n = 574	*P* value
Age (years)			0.149
≤44	46(15.23)	117(20.38)	
45-59	143(47.35)	279(48.61)	
60-74	87(28.81)	137(23.87)	
≥75	26(8.61)	41(7.14)	
Gender			0.104
Male	200(66.23)	348(60.63)	
Female	102(33.77)	226(39.37)	
Education Level			<0.001
Illiteracy	40(13.25)	71(12.37)	
Primary-Middle school	189(62.58)	256(44.60)	
High school or above	73(24.17)	247(43.03)	
Marital status			0.113
Married	268(88.74)	528(91.99)	
Others	34(11.26)	46(8.01)	
Residence			<0.001
Rural	154(50.99)	161(28.05)	
Urban	148(49.01)	413(71.95)	
Family history of cancer			0.660
No	235(77.81)	454(79.09)	
Yes	67(22.19)	120(20.91)	
Tobacco smoking			<0.001
No	143(47.35)	408(71.08)	
Yes	159(52.65)	166(28.92)	
Alcohol drinking			<0.001
No	179(59.27)	459(79.97)	
Yes	123(40.73)	115(20.03)	
Vegetables			<0.001
≤1 time/day	139(46.03)	168(29.27)	
>1times/day	163(53.97)	406(70.73)	
Fruits			<0.001
≤3 times/week	233(77.15)	276(48.08)	
>3 times/week	69(22.85)	298(51.92)	

**Figure 1:**
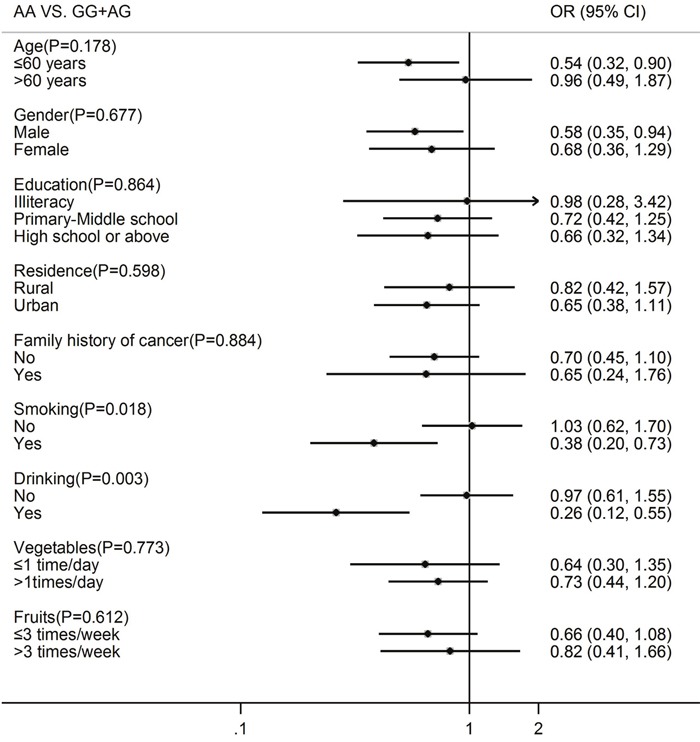
*FADS1* rs174549 polymorphism and the risk of oral cancer stratified by demographics and main lifestyle factors

Table 2 shows the effects of rs174549 polymorphism and fish consumption on oral cancer. Genotype distribution of *FADS1* (rs174549) among controls was in agreement with Hardy-Weinberg equilibrium (*P*>0.05). After adjustment for potential confounders, *FADS1* A variant allele was associated with a significantly decreased risk of oral cancer: the ORs were 0.65 (95% CI: 0.42-0.99) for codominant model and 0.67 (95% CI: 0.46-0.98) for recessive model. Moreover, when stratified by demographic characteristics, the statistically significant reverse associations were only emerged in men and those age ≤ 60 years. When stratified by main lifestyle factors, the protective effect of AA genotype was especially evident in smokers and alcohol drinkers (Figure 1).

**Table 2:** Effects of *FADS1* rs174549 polymorphism and fish intake on oral cancer

Variable	Case (%) (n = 302)	Control (%) (n = 574)	Unadjusted odds ratios (95% CI)	Adjusted odds ratios^a^ (95% CI)
rs1745496 (P_HWE_=0.32)				
Codominant model				
GG	106(35.10)	169(29.44)	1.00	1.00
AG	147(48.67)	274(47.74)	0.86(0.62-1.17)	0.86(0.62-1.20)
AA	49(16.23)	131(22.82)	0.60(0.40-0.90)	0.65(0.42-0.99)
Dominant model				
GG	106(35.10)	169(29.44)	1.00	1.00
AG+AA	196(64.90)	405(70.56)	1.30(0.96-1.74)	1.26(0.92-1.72)
Recessive model				
GG+AG	253(83.77)	443(77.18)	1.00	1.00
AA	49(16.23)	131(22.82)	0.65(0.46-0.94)	0.67(0.46-0.98)
Fish intake				
0-2 times/week	138(45.69)	147(25.61)	1.00	1.00
3-6 times/week	112(37.09)	178(31.01)	0.67(0.48-0.93)	0.85(0.58-1.25)
≥7 times/week	52(17.22)	249(43.38)	0.22(0.15-0.32)	0.27(0.18-0.42)
P for trend			<0.001	<0.001

^a^ Adjusted for age, gender, education, marital status, residence, family cancer history, smoking, drinking, vegetables and fruits.

Additionally, with regard to fish consumption, the frequency of fish intake was categorized into three groups according to the tertiles of controls (0-2 times/week, 3-6 times/week, ≥7 times/week). Fish intake ≥7 times/week showed a 73% reduction in risk for oral cancer compared to those who ate fish less than 2 times/week (OR: 0.27, 95% CI: 0.18-0.42). Moreover, there was a tendency of decreased risk with the increasing frequency of fish consumption (all P for trend <0.001).

We further evaluated the joint effects of rs174549 polymorphism in recessive model and fish consumption on the risk of oral cancer (Table 3). A significantly lower OR was observed in individuals who carrying AA genotype and consumed fish ≥7times/week compared with GG+AG carriers who ate fish less than 2 times/week (OR: 0.30, 95% CI: 0.14-0.63). Moreover, a positive multiplicative interaction between *FADS1* gene and fish intake for oral cancer was found (OR_multiplicative_ = 0.70, 95% CI: 0.51-0.96, *P*=0.028; data not shown).

**Table 3:** Interactions between *FADS1* rs174549 polymorphism and fish intake in oral cancer

Variables		Cases (%)N =302	Controls (%)N = 574	OR (95% CI)	OR^a^ (95% CI)
FADS1	Fish intake				
GG+AG	0-2 times/week	120(39.74)	120(20.91)	1.00	1.00
GG+AG	3-6 times/week	94(31.13)	124(21.60)	0.76(0.52-1.10)	0.93(0.60-1.42)
GG+AG	≥7times/week	39(12.91)	199(34.67)	0.20(0.13-0.30)	0.25(0.15-0.40)
AA	0-2 times/week	18(5.96)	27(4.70)	0.67(0.35-1.27)	0.74(0.36-1.52)
AA	3-6 times/week	18(5.96)	54(9.41)	0.33(0.18-0.60)	0.50(0.25-0.97)
AA	≥7times/week	13(4.30)	50(8.71)	0.26(0.13-0.50)	0.30(0.14-0.63)

^a^ Adjusted for age, gender, education, marital status, residence, family cancer history, smoking, drinking, vegetables and fruits.

## DISCUSSION

To our knowledge, this case-control study is the first to report independent and joined effects of the new susceptibility loci in *FADS1* (rs174549) and fish consumption on oral cancer in southeast China. We found AA genotype was associated with a decreased risk of oral cancer compared to the GG genotype. Moreover, fish intake ≥7times/week also reduced the risk of oral cancer. Furthermore, there was a positive multiplicative interaction between *FADS1* gene and fish intake for oral cancer.

To date, only a GWAS study revealed that *FADS1* polymorphism (rs174549) showed a protective effect on LSCC [8]. This finding is consistent with the present results. Although the mechanism of rs174549 polymorphism on oral cancer is not clear, previous study found there were 49 SNPs in high linkage disequilibrium with rs174549 in the same chromosome, and these SNPs might influence the expression of *FADS1* through their effects on host genes [8]. Moreover, *FADS1* variation could suppress inflammatory response through influencing the metabolism of PUFAs. The main metabolites of PUFAs include arachidonic acid (AA; a pro-inflammatory factor), eicosapentaenoic acid (EPA), and docosahexaenoic acid (DHA) (EPA and DHA are anti-inflammatory factors). Horiguchi et al.[16] found *FADS1* polymorphism (rs174547) was correlated with lower AA, but unchanged for EPA or DHA. Yao et al.[17] showed *FADS1-FADS2* gene cluster variation could inhibit the conversion of α-linolenic acid (ALA) to AA. Tanaka et al.[18] demonstrated rs174537 polymorphism in *FADS1* could increase the expression of EPA. Therefore, we speculated that imbalance between AA and EPA and DHA might be a mechanism of *FADS1* rs174549 polymorphism on oral cancer.

Our study revealed that fish intake might be a benefit factor for oral cancer risk, which is consistent with previous studies [19]. Higher intakes of fish which contain key anti-inflammatory nutrients (LC-PUFA, EPA, DHA, etc.)[20], have been reported to suppress inflammation, oxidative stress and cancer risk in animal study [21] and observational study [22]. Additionally, Actis et al.[23] found dietary lipids (especially for n-3 fatty acids) also reduced cell proliferation and differentiation of murine oral squamous epithelium. Therefore, these have been reasonably hypothesized that fish consumption may lower the risk of oral cancer.

Interestingly, our results demonstrated a significant gene-diet interaction between *FADS1* gene and fish intake for oral cancer risk. Yeates et al.[24] showed that *FADS1* gene variant was associated with decreased level of AA in serum and increased ALA to DHA in high fish intakes population. An explanation for our finding might be that the genetic variation in *FADS1* could interact with dietary fish oil to increase delta-5 activity and change LC-PUFA proportions [25].

There are some limitations in this study. First, the present study only evaluated the effect of total fish consumption on oral cancer, and did not further to analyze the effects of different fish species and the preparation methods. Hence, these factors should be taken into account in future studies. Second, only one SNP was chosen based on a recent GWAS which may not represent a comprehensive view of *FADS1* gene variation. Further studies on variation in susceptible regions of *FADS1* gene are needed. Third, since this is a very preliminary study, further animal or cell experiments with more rigorous design are also required to explore the mechanisms.

In conclusion, our results suggest that *FADS1* rs174549 polymorphism showed a protective role in etiology of oral cancer. Moreover, fish intake may be an interacting factor that decreases oral cancer risk in individuals with the mutant genotype of rs174549. Further research on gene-diet interaction in oral cancer is warranted to obtain more conclusive outcomes.

## MATERIALS AND METHODS

### Study design and population

This hospital-based case-control study was conducted from September 2005 to September 2010 in Fujian, China. 305 oral cancer patients were recruited from the First Affiliated Hospital of Fujian Medical University. As reported previously [26], inclusion criteria of cases were as follows: (1) all cases were newly diagnosed and histologically confirmed primary oral cancer; (2) all cases are Chinese Han population and live in Fujian Province; (3) all cases aged 20 to 80 years. Patients with second primary, recurrent oral cancer, previous radiotherapy or chemotherapy, were excluded from this study.

579 cancer-free control subjects were selected from the physical examination population in the same hospital and frequency-matched to the cases group by gender and age (±3 years). The cancer-free status was ascertained according to the results of physical examination. Those who were direct relatives to the cases or had a previous history of cancer were excluded. The recruiting rate for oral cancer patients was 98.3% and the rate for control subjects was 96.9%. The present study was approved by the Institutional Review Board (IRB) of Fujian Medical University (Fuzhou, China). All participants agreed to this study and signed a consent form.

### Data and sample collections

All epidemiological data were collected by face-to-face interview using a standardized questionnaire, including information on demographic characteristics, smoking, drinking, diet factors, residential history, and family history of cancer. The subjects were considered smokers if they had smoked at least 100 cigarettes during their lifetime. Alcohol consumers were defined as those who had consumed at least 1 drink/week continuously for at least 6 months. A 5-10 ml blood sample was collected from each subjects with an EDTA-coated vacuum tube and stored at −80°C.

### Selection of SNPs and genotyping

Genomic DNA was extracted from whole-blood samples using the Qiagen Blood Kit (Qiagen, Chatsworth, CA). All samples were genotyped by the 50-nuclease TaqMan assay, using the ABI PRISM 7900HT Sequence Detection System (ABI, Foster City, CA). TaqMan primers and FAM- or VIC-labeled probes were designed using the Primer Express Oligo Design software v2.0 (ABI PRISM). The PCR primers were as follows: forward 5’-CCAGCCTGTCTACTTTCCCA-3’ and reverse 5’-TCTACGTCCGCT TCTTCCTCAC-3’. Amplification conditions were as follows: 95°C for 10 min, then followed by 40 cycles of 95°C for 5 sec, and 60°C for 30 sec. Allelic Discrimination Sequence Detector Software was used to read the completed PCR plates with an ABI 7900HT Sequence Detector in the end point mode. For the assessment of genotyping results, laboratory personnel were blinded to the case-control status. All assays were carried out in 384-well arrays with 8 no-template controls and 8 duplicated samples in each plate for quality control. Approximately 5% of the samples were randomly repeated for quality control purposes. Genotyping call rates were over 99.0% and the concordance rate reached 100%. Due to genotyping failure of some DNA samples, only 302 cases and 574 control subjects with complete genotyping data can be used for further analysis.

### Statistical analysis

Statistical software R (version 3.1.1) was used for our data analyses. The χ^2^ test was performed for the socio-demographic covariates of the case and control subjects. Hardy–Weinberg equilibrium was conducted using a goodness-of-fit χ^2^ test with linkage disequilibrium analyzer (LDA) software v.1.0 for SNP among the controls. Unconditional logistic regression models were used to estimate odds ratios (ORs) and their 95% confidence intervals (CIs). Interaction between the SNP and fish intake was evaluated using unconditional logistic regression model. Statistical significance was considered at the *P*<0.05 level.
